# Early biopsychological changes during masculinizing gender-affirming hormone therapy in AFAB transgender individuals: a 4-month prospective study

**DOI:** 10.3389/fpsyt.2026.1847778

**Published:** 2026-06-17

**Authors:** Ludek Fiala, Daniela Kestlerova, Jakub Nespor, Jiri Lenz

**Affiliations:** 1 Psychiatric Clinic, Department of Sexology, Faculty of Medicine, Charles University, Pilsen, Czechia; 2 Institute of Sexology, Faculty Hospital, First Faculty of Medicine, Charles University, Prague, Czechia; 3 Department of Pathology, Znojmo Hospital, Znojmo, Czechia; 4 Department of Anatomy, Histology and Embryology, Faculty of Veterinary Medicine, University of Veterinary and Pharmaceutical Sciences Brno, Brno, Czechia

**Keywords:** anxiety, gender-affirming hormone therapy, quality of life, transgender men, trauma-related symptoms

## Abstract

**Objective:**

Transgender individuals frequently experience elevated levels of psychological distress, including anxiety and trauma-related symptoms. Gender-affirming hormone therapy (GAHT) has been associated with improvements in mental health and quality of life; however, early biopsychological changes occurring during the initial phase of masculinizing hormone therapy remain insufficiently understood. The present exploratory longitudinal study investigated early endocrine and psychological changes in transgender individuals assigned female at birth undergoing masculinizing GAHT.

**Methods:**

We conducted a 4-month prospective observational study involving 30 transgender individuals assigned female at birth initiating masculinizing GAHT. Participants were assessed at baseline (prior to treatment initiation), after 2 months, and after 4 months of therapy. Hormonal parameters (estradiol, testosterone, prolactin) and psychological measures including anxiety (HAM-A), trauma-related symptoms (TSC-40), and quality of life (MANSA) were evaluated. Due to the exploratory character of the study and the relatively small sample size, analyses were performed primarily using non-parametric statistical methods, including repeated-measures analyses.

**Results:**

During follow-up, expected endocrine changes were observed, including decreased estradiol levels and increased testosterone concentrations. These changes were accompanied by reductions in anxiety and trauma-related symptoms and by improvements in quality of life measures over time. Repeated-measures analyses demonstrated significant longitudinal improvements in psychological outcomes across follow-up assessments (p < 0.05). Exploratory associations between hormonal and psychological variables appeared more pronounced after 4 months of treatment, particularly for estradiol in relation to anxiety and quality of life. Earlier assessments demonstrated weaker and less consistent associations. Psychological improvements were not directly proportional to the magnitude of hormonal changes alone, suggesting that psychosocial and treatment-related factors may also contribute to early adaptation during GAHT.

**Conclusion:**

Early masculinizing GAHT in transgender individuals assigned female at birth was associated with favorable endocrine and psychological changes during the first 4 months of treatment. The findings support a complex and potentially time-dependent relationship between hormonal alterations and psychological adaptation during early transition. Given the exploratory nature and limited sample size of the present study, further longitudinal research with larger cohorts and comparison groups is needed to clarify the underlying biopsychological mechanisms of GAHT.

## 1 Introduction

Transgender individuals frequently experience elevated levels of psychological distress, including anxiety, depressive symptoms, and trauma-related symptomatology. These difficulties are commonly interpreted within the framework of minority stress theory, which proposes that chronic exposure to stigma, discrimination, social rejection, and internalized negative societal attitudes contributes to sustained psychophysiological stress and adverse mental health outcomes (1).

In recent years, many clinical centers have reported an increasing number of individuals assigned female at birth seeking masculinizing transition. The factors contributing to this increase are likely multifactorial and may involve biological, psychological, and sociocultural influences. Prior to transition, many individuals experience significant gender dysphoria associated with incongruence between experienced gender identity and assigned sex, often accompanied by body-related distress, social discomfort, and identity-related conflict. Previous studies have also documented elevated rates of interpersonal trauma, social rejection, and minority stress among transgender populations, contributing to increased prevalence of anxiety disorders, depressive symptoms, and trauma-related syndromes.

From a neuroendocrine perspective, sex hormones may modulate emotional regulation, stress responsivity, and limbic system functioning. Testosterone administration has been associated with changes in emotional processing, stress reactivity, and social behavior, potentially contributing to improved psychological adaptation in some transgender individuals undergoing masculinizing hormone therapy (2). However, these effects appear complex and may be influenced by pre-existing psychological burden, trauma history, psychosocial adaptation, and environmental context.

Gender-affirming hormone therapy (GAHT) therefore represents not only a somatic intervention but also a complex biopsychosocial process. Previous studies have documented elevated levels of minority stress, discrimination, trauma-related burden, and mental health disparities among transgender populations (3, 4). Nevertheless, the early dynamics of endocrine and psychological changes during the initial phase of masculinizing hormone therapy remain insufficiently characterized, particularly regarding trauma-related symptoms and quality of life.

The present exploratory longitudinal study aimed to evaluate hormonal changes, anxiety, trauma-related symptoms, and quality of life in transgender individuals assigned female at birth undergoing masculinizing GAHT during the first four months of treatment. In addition, we sought to examine associations between endocrine variables and psychological outcomes over time in order to better understand potential biopsychological mechanisms associated with early treatment adaptation.

## 2 Methods

### 2.1 Study design and participants

This prospective observational study included 30 transgender individuals assigned female at birth undergoing masculinizing GAHT. Participants were consecutively recruited at a specialized clinical center between January and October 2025 and were all 18 years of age or older at the time of enrollment. Assessments were performed at three time points: prior to initiation of hormone therapy (baseline), after 2 months of treatment, and after 4 months of treatment.

All participants met clinical diagnostic criteria for gender dysphoria and were indicated for masculinizing hormone therapy according to standard clinical guidelines. The mean age of participants was 21.7 years (SD = 6.06). All participants were Czech-speaking and completed validated Czech versions of all psychometric instruments.

No participants had undergone previous unilateral or bilateral oophorectomy or any gender-affirming surgical procedure prior to or during the study period. No participants used hormonal contraceptives or adjunctive endocrine therapies such as 5-alpha reductase inhibitors. No clinically significant non-adherence to hormone therapy was identified during follow-up.

### 2.2 Hormone therapy

Gender-affirming hormone therapy consisted of testosterone administration according to current clinical practice. Treatment regimens were individualized based on clinical evaluation and regularly monitored by an experienced endocrinologist. Masculinizing hormone therapy was administered using a depot formulation of mixed testosterone esters applied intramuscularly at approximately three-week intervals in accordance with standard clinical practice.

### 2.3 Measures

Hormonal parameters included serum levels of estradiol, testosterone, and prolactin. Blood samples were collected in the morning under standard clinical conditions and analyzed according to the methodology of the Department of Clinical Biochemistry, University Hospital Plzeň.

Psychological assessment included the Hamilton Anxiety Rating Scale (HAM-A) (5), the Trauma Symptom Checklist (TSC-40) (6), and the Manchester Short Assessment of Quality of Life (MANSA) (7). All instruments are internationally validated, adapted for the Czech language, and routinely used in clinical and research practice.

Menstrual cycle phase was not systematically controlled for during assessments. Given the psychological discomfort associated with menstruation in many participants gender-affirming treatment, cycle monitoring was intentionally kept minimal to reduce participant burden.

### 2.4 Procedure

All participants underwent comprehensive baseline evaluation, including clinical, hormonal, and psychological assessment. Follow-up assessments were performed after 2 and 4 months of hormone therapy using the same measurement protocol.

### 2.5 Statistical analysis

Descriptive statistics were used to summarize sample characteristics and measured variables. Continuous variables are presented as means and standard deviations. Changes over time were analyzed using non-parametric repeated-measures tests (Friedman ANOVA). Associations between hormonal and psychological variables were evaluated using Spearman’s rank correlation coefficients.

Given the exploratory character of the study and the relatively small sample size, conservative non-parametric analytical approaches were preferred over more complex longitudinal modeling strategies. Correlation analyses were considered exploratory and hypothesis-generating. Statistical significance was set at p < 0.05. Analyses were performed using Statistica software (Statistica version 12).

## 3 Results

### 3.1 Sample characteristics

The study included 30 transgender individuals assigned female at birth undergoing masculinizing GAHT. All participants completed baseline, 2-month, and 4-month follow-up assessments. Demographic and clinical characteristics of the study sample are summarized in **Table 1**.

**Table 1:** Participant characteristics.

Variable	Value
Participants	30
Age (mean ± SD)	21.7 ± 6.1
Czech-speaking	100%
Previous GA surgery	0
Hormonal contraception	0
Oophorectomy	0

### 3.2 Hormonal changes over time

Progressive endocrine changes consistent with masculinizing hormone therapy were observed during follow-up. Estradiol concentrations gradually decreased, whereas testosterone concentrations increased over the 4-month treatment period. Prolactin levels demonstrated moderate variability over time.

Detailed hormonal findings across follow-up assessments are presented in **Table 2**.

**Table 2:** Hormonal parameters over time.

Parameter	Baseline mean ± SD	2 Months mean ± SD	4 Months mean ± SD	Friedman p
Estradiol	343.1 ± 142.3	251.3 ± 119.8	189.1 ± 79.0	p < 0.001
Testosterone	1.03 ± 0.55	6.95 ± 1.77	11.32 ± 2.07	p < 0.001
Prolactin	296.3 ± 83.2	286.2 ± 78.6	276.5 ± 70.3	n.s.

Friedman repeated-measures analyses demonstrated significant longitudinal reductions in estradiol concentrations and significant increases in testosterone concentrations during follow-up (both p < 0.001). Changes in prolactin concentrations were not statistically significant.

### 3.3 Changes in psychological measures

Psychological measures demonstrated gradual improvement during follow-up. Anxiety symptoms assessed using HAM-A decreased over time, while trauma-related symptoms measured by TSC-40 also showed reduction. Subjective quality of life assessed by MANSA progressively increased from baseline to the 4-month follow-up.

Detailed longitudinal changes in psychological measures are summarized in **Table 3**.

**Table 3:** Psychological outcomes over time.

Measure	Baseline mean ± SD	2 months mean ± SD	4 months mean ± SD	Friedman p
HAM-A	21.0 ± 5.0	20.2 ± 4.9	17.5 ± 4.2	p < 0.01
TSC-40	75.7 ± 14.3	70.3 ± 11.6	69.5 ± 11.8	p < 0.05
MANSA	22.1 ± 5.0	23.1 ± 3.9	26.2 ± 5.8	p < 0.01

Friedman repeated-measures analyses demonstrated statistically significant longitudinal improvements in HAM-A, TSC-40, and MANSA scores across the study period (all p < 0.05).

### 3.4 Exploratory correlation analyses

Exploratory correlation analyses were performed to evaluate associations between endocrine and psychological variables across follow-up assessments.

At baseline, weak negative associations were observed between estradiol (E2) and prolactin (PRL) (ρ = −0.47, p < 0.05) and between E2 and trauma-related symptoms (TSC-40) (ρ = −0.40, p < 0.05).

After 2 months of treatment, moderate associations were identified between E2 and total testosterone (TT) (ρ = −0.47, p < 0.05), between E2 and anxiety (HAM-A) (ρ = 0.40, p < 0.05), and between anxiety (HAM-A) and trauma-related symptoms (TSC-40) (ρ = 0.39, p < 0.05).

After 4 months of treatment, several exploratory associations emerged. Unexpected positive associations between estradiol levels and psychological measures were observed after 4 months of treatment, specifically between E2 and quality of life (MANSA) (ρ = 0.67, p < 0.05) and between E2 and anxiety (HAM-A) (ρ = 0.63, p < 0.05). Total testosterone (TT) was positively associated with quality of life (MANSA) (ρ = 0.58, p < 0.05).

Overall, the strongest endocrine-psychological associations were observed at the 4-month follow-up, which may suggest time-dependent biopsychological adaptation during early masculinizing hormone therapy. Given the exploratory character of the analyses and the relatively small sample size, these findings should be interpreted cautiously.

### 3.5 Figure references

**Tables 1**–**3** illustrate exploratory associations between selected hormonal and psychological variables at baseline, after 2 months, and after 4 months of treatment.

## 4 Discussion

The present pilot longitudinal study examined early biopsychological changes during the first four months of gender-affirming hormone therapy (GAHT) in transgender individuals assigned female at birth undergoing masculinizing treatment. The findings indicate that initiation of masculinizing GAHT was accompanied by expected endocrine changes together with gradual improvements in anxiety, trauma-related symptoms, and subjective quality of life. These observations are generally consistent with previous studies reporting improvements in psychological well-being during gender-affirming treatment in transgender populations. The longitudinal trends observed in both hormonal and psychological variables may support gradual biopsychological adaptation during early treatment.

An important finding of the present study is the apparent time-dependent character of biopsychological adaptation during early treatment. Although some psychological improvement was already observable after two months of therapy, the strongest associations between hormonal and psychological variables emerged after four months of follow-up. This pattern suggests that psychological adaptation during GAHT may not occur immediately after endocrine changes but may instead develop progressively over time. Such delayed effects are consistent with previous observations indicating that psychological benefits associated with GAHT often emerge gradually during ongoing treatment and psychosocial adjustment (4).

From a neurobiological perspective, sex hormones may influence emotional regulation, stress responsivity, and limbic system functioning. Testosterone administration has previously been associated with changes in emotional processing, social behavior, and stress reactivity (8). Nevertheless, the associations observed in the present study were not entirely uniform, and some findings — particularly the unexpected positive association between estradiol and anxiety at 4 months — indicate that these relationships are likely complex and influenced by multiple interacting factors. Interindividual differences in hormonal sensitivity, prior psychological burden, psychosocial stress exposure, and environmental context may all contribute to variability in psychological adaptation during early transition.

Importantly, the observed reductions in trauma-related symptoms and anxiety may not be attributable solely to endocrine changes themselves. Initiation of gender-affirming treatment may also contribute to psychological relief through reduction of gender dysphoria, increased congruence between experienced gender identity and bodily characteristics, and improved expectations regarding social functioning and future identity integration. The present findings therefore support a broader biopsychosocial interpretation of treatment effects rather than a purely hormonal explanation.

At the same time, the partially inconsistent pattern of correlations between hormonal and psychological variables further supports the importance of adopting a biopsychosocial framework (9). Hormonal changes alone are unlikely to fully explain psychological outcomes gender-affirming treatment. Instead, treatment effects are probably mediated by a dynamic interaction of endocrine factors, minority stress, prior trauma exposure, resilience, interpersonal support, and broader social adaptation processes (10).

The present study focused exclusively on individuals undergoing masculinizing transition. This decision was made in order to study a relatively homogeneous cohort during the initial phase of masculinizing hormone therapy and to reduce heterogeneity related to differing endocrine regimens and psychosocial adaptation trajectories between masculinizing and feminizing hormone therapy populations. At the same time, we acknowledge that many psychological stressors affecting transgender individuals are shared across both groups. Importantly, longitudinal recruitment of individuals undergoing male-to-female (M→F) transition is currently ongoing at our institution, which may allow future comparative analyses between both transgender populations.

From a clinical perspective, the findings emphasize the importance of integrated multidisciplinary care during early transition (11). Although GAHT represents a central component of treatment for gender dysphoria, psychological and psychotherapeutic support focused on stress regulation, trauma processing, identity integration, and social adaptation may remain equally important, particularly during the initial phases of gender-affirming treatment when substantial biological and psychosocial changes occur simultaneously.

Several limitations should be considered when interpreting the present findings. First, the sample size was relatively small, reflecting the exploratory and pilot character of the study, and the follow-up period was limited to four months. Second, the observational design precludes causal interpretation of the observed associations. Third, menstrual cycle phase was not systematically controlled prior to treatment initiation, which may have contributed to variability in estradiol levels. Fourth, psychosocial variables such as social support, life events, or ongoing psychotherapy were not systematically assessed. The absence of a control group further limits interpretation of endocrine-psychological associations. Finally, correlation analyses were exploratory in nature and were not adjusted for multiple comparisons; therefore, findings should be interpreted cautiously and require confirmation in larger longitudinal studies.

## 5 Conclusion

The present pilot longitudinal study provides preliminary insight into early endocrine and psychological changes during masculinizing gender-affirming hormone therapy in transgender individuals assigned female at birth. Improvements in anxiety, trauma-related symptoms, and quality of life were observed during the first four months of treatment, while associations between hormonal and psychological variables appeared to strengthen over time.

At the same time, the findings suggest that psychological improvement during early GAHT is not directly proportional to the magnitude of estradiol reduction or testosterone increase alone. Rather, initiation and continuation of gender-affirming treatment may represent part of a broader biopsychosocial adaptation process involving endocrine, psychological, and social mechanisms.

Given the exploratory nature of the study, relatively small sample size, and absence of a comparison group, the findings should be interpreted cautiously. Future longitudinal studies with larger cohorts, extended follow-up periods, and inclusion of individuals undergoing male-to-female (M→F) transition will be necessary to better clarify the differential biopsychological trajectories associated with gender-affirming hormone therapy.

## Data Availability

The original contributions presented in the study are included in the article/supplementary material. Further inquiries can be directed to the corresponding author.

## References

[1] Lefevor GT Boyd-Rogers CC Sprague BM Janis RA . Health disparities between genderqueer, transgender, and cisgender individuals: An extension of minority stress theory. J Couns Psychol. (2019) 66:385–95. doi: 10.1037/cou0000339 30896208

[2] D’hoore L T’Sjoen G . Gender-affirming hormone therapy: An updated literature review with an eye on the future. J Internal Med. (2022) 291:574–92. doi: 10.1111/joim.13441 34982475

[3] Reisner SL White Hughto JM Gamarel KE Keuroghlian AS Mizock L Pachankis JE . Discriminatory experiences associated with posttraumatic stress disorder symptoms among transgender adults. J Couns Psychol. (2016) 63:509–19. doi: 10.1037/cou0000143 26866637 PMC4981566

[4] White Hughto JM Murchison GR Clark K Pachankis JE Reisner SL . Geographic and individual differences in healthcare access for U.S. transgender adults: A multilevel analysis. LGBT. Health. (2016) 3:424–33. doi: 10.1089/lgbt.2016.0044 27636030 PMC5165678

[5] Hamilton M . The assessment of anxiety states by rating. Br J Med Psychol. (1959) 32:50–5. doi: 10.1111/j.2044-8341.1959.tb00467.x 13638508

[6] Briere J . Dissociative symptoms and trauma exposure: Specificity, affect dysregulation, and posttraumatic stress. J Nervous. Ment Dis. (2006) 194:78–82. doi: 10.1097/01.nmd.0000198139.47371.54 16477184

[7] Priebe S Huxley P Knight S Evans S . Application and results of the manchester short assessment of quality of life (MANSA). Int J Soc Psychiatry. (1999) 45:7–12. doi: 10.1177/002076409904500102 10443245

[8] Tordoff DM Wanta JW Collin A Stepney C Inwards-Breland DJ Ahrens K . Mental health outcomes in transgender and nonbinary youths receiving gender-affirming care. JAMA Netw Open. (2022) 5:e220978. doi: 10.1001/jamanetworkopen.2022.0978 35212746 PMC8881768

[9] Engel GL . The need for a new medical model: A challenge for biomedicine. Science. (1977) 196:129–36. doi: 10.1126/science.847460 847460

[10] Bockting WO Miner MH Swinburne Romine RE Hamilton A Coleman E . Stigma, mental health, and resilience in an online sample of the US transgender population. Am J Public Health. (2013) 103:943–51. doi: 10.2105/AJPH.2013.301241 23488522 PMC3698807

[11] Coleman E Radix AE Bouman WP Brown GR de Vries ALC Deutsch MB . Standards of care for the health of transgender and gender diverse people, version 8. Int J Transgender. Health. (2022) 23:S1–S259. doi: 10.1080/26895269.2022.2100644 36238954 PMC9553112

